# Impact of assimilable nitrogen availability in glucose uptake kinetics in *Saccharomyces cerevisiae* during alcoholic fermentation

**DOI:** 10.1186/1475-2859-11-99

**Published:** 2012-07-30

**Authors:** Margarida Palma, Sara Cordeiro Madeira, Ana Mendes-Ferreira, Isabel Sá-Correia

**Affiliations:** 1 Institute for Biotechnology and Bioengineering, Centre for Biological and Chemical Engineering, Instituto Superior Técnico, Technical University of Lisbon, 1049-001 Lisbon, Portugal; 2 Department of Bioengineering, Instituto Superior Técnico, Technical University of Lisbon, 1049–001, Lisbon Portugal; 3 Knowledge Discovery and Bioinformatics (KDBIO) Group, INESC-ID, Lisbon, Portugal; 4 Department of Computer Science and Engineering, Instituto Superior Técnico, Technical University of Lisbon, 1049-001, Lisboa, Portugal; 5 Institute for Biotechnology and Bioengineering, Centro de Genómica e Biotecnologia, Universidade de Trás-os-Montes e Alto Douro, Vila Real, Portugal

**Keywords:** Hexose transporters, Glucose uptake kinetics, Alcoholic fermentation, Nitrogen regulation, Yeast, Biclustering analysis

## Abstract

**Background:**

The expression and activity of the different *Saccharomyces cerevisiae* hexose uptake systems (Hxt) and the kinetics of glucose uptake are considered essential to industrial alcoholic fermentation performance. However, the dynamics of glucose uptake kinetics during the different stages of fermentation, depending on glucose and nitrogen availability, is very poorly characterized. The objective of the present work was to examine thoroughly the alterations occurring in glucose uptake kinetics during alcoholic fermentation, by the wine strain *S. cerevisiae* PYCC 4072, of a synthetic grape juice basal medium with either a limiting or non-limiting initial nitrogen concentration and following nitrogen supplementation of the nitrogen-depleted sluggish fermentation.

**Results:**

Independently of the initial concentration of the nitrogen source, glucose transport capacity is maximal during the early stages of fermentation and presumably sustained by the low-affinity and high-capacity glucose transporter Hxt1p. During nitrogen-limited sluggish fermentation, glucose uptake capacity was reduced to approximately 20% of its initial values (*V*_max_ = 4.9 ± 0.8 compared to 21.9 ± 1.2 μmol h^-1^ 10^-8^ cells), being presumably sustained by the low-affinity glucose transporter Hxt3p (considering the calculated *K*_m_ = 39.2 ± 8.6 mM). The supplementation of the sluggish fermentation broth with ammonium led to the increase of glucose transport capacity associated to the expression of different glucose uptake systems with low and high affinities for glucose (*K*_m_ = 58.2 ± 9.1 and 2.7 ± 0.4 mM). A biclustering analysis carried out using microarray data, previously obtained for this yeast strain transcriptional response to equivalent fermentation conditions, indicates that the activation of the expression of genes encoding the glucose transporters Hxt2p (during the transition period to active fermentation) and Hxt3p, Hxt4p, Hxt6p and Hxt7p (during the period of active fermentation) may have a major role in the recovery of glucose uptake rate following ammonium supplementation. These results suggest a general derepression of the glucose-repressible HXT genes and are consistent with the downregulation of Mig1p and Rgt1p.

**Conclusions:**

Although reduced, glucose uptake rate during nitrogen-limited fermentation is not abrogated. Following ammonium supplementation, sluggish fermentation recovery is associated to the increase of glucose uptake capacity, related to the *de novo* synthesis of glucose transporters with different affinity for glucose and capacity, presumably of Hxt2p, Hxt3p, Hxt4p, Hxt6p and Hxt7p. This study is a contribution to the understanding of yeast response to different stages of alcoholic fermentation at the level of glucose uptake kinetics, in particular under nitrogen limitation or replenish, which is useful knowledge to guide fermentation practices.

## Background

The accumulation of inhibitory metabolites during alcoholic fermentation (e.g. ethanol, acetic acid, long chain fatty acids, etc.) and nutritional deficiencies are considered the main causes of stuck or sluggish fermentations conducted by *Saccharomyces cerevisiae*[1-4]. The availability of assimilable nitrogen, whose abundance is variable in the natural substrates used for alcoholic fermentation, influences yeast cells performance during the process. The assimilable nitrogen content in grape juice can range from 60 to 2400 mg L^-1^[3]; for levels below 120–140 mg L^-1^, a nitrogen supplementation, usually as diammonium phosphate, is needed to avoid wine fermentation arrest before the complete fermentation of the high sugar concentrations present [3]. The production of other alcoholic beverages, such as beer and the Brazilian spirit *cachaça*, or of bioethanol from different substrates can also be improved by media supplementation with an assimilable nitrogen source [5-7]. Indeed, it has been proposed that the major limiting factor of enological fermentation occurring upon nitrogen depletion is the inactivation of sugar transporters associated to protein synthesis arrest and their degradation, even when glucose concentration is still high [8-11].

Sugar transport across *S. cerevisiae* plasma membrane is carried out by a complex and highly regulated system of eighteen hexose permeases, from Hxt1p to Hxt17p and Gal2p. The genes *HXT1-HXT4* and *HXT6,7* are thought to encode the major Hxt proteins since their individual expression is able to restore growth on glucose in a mutant strain deleted for *HXT1-7*[12]. The different hexose transporters are transcriptionally regulated in response to glucose availability. For example, the genes encoding low-affinity glucose transporters are activated at high concentrations of glucose (e.g. *HXT1*) [13,14], or constitutively expressed in the presence of glucose (e.g. *HXT3*) [14], while the transcription of genes encoding the high-affinity glucose transporters, Hxt6p and Hxt7p is activated when glucose is scarce in the medium [15,16]. *HXT2* and *HXT4* encode proteins with a moderate/high affinity for glucose [12,17], both induced by low levels of glucose and repressed by high levels of glucose, although *HXT2* transcription can also be repressed by a different regulatory mechanism when glucose is absent [14]. *HXT5,* which codes for a moderate-affinity glucose transporter, is not expressed during yeast growth in rich medium with glucose [18], but is transcriptionally activated under conditions leading to low specific growth rate, such as under nitrogen limitation [19-21]. Under specific conditions, the hexose permeases are marked for degradation via endocytosis and transported for degradation in the vacuole [22-24]. For instance, Hxt6p and Hxt7p are internalized by endocytosis under nitrogen starvation when a fermentable carbon source is present [25].

The activity of hexose uptake systems has been considered the factor that limits alcoholic fermentation activity but the variation of sugar uptake kinetics throughout nitrogen-limited sluggish fermentation and the same replenished fermentation, is poorly characterized. The objective of the present work was to examine thoroughly the alteration occurring in glucose uptake kinetics during alcoholic fermentation by the wine strain *S. cerevisiae* PYCC 4072 of a synthetic grape juice basal medium with either a limiting or non-limiting initial nitrogen concentration and following nitrogen supplementation of the nitrogen-depleted sluggish fermentation. The three different nitrogen availability conditions tested in the present work are similar to those described before [26]. The reanalysis of the gene transcription profiles obtained for the same yeast strain during fermentations carried out under similar nitrogen supply conditions was also carried out by exploring a biclustering algorithm for identification of groups of genes with similar expression patterns in a time series gene expression data [27]. This biclustering analysis provided clues into the alterations occurring at the expression level of hexose transporters encoding genes and their putative transcription regulators during the various fermentation conditions. In particular, the selected biclusters corresponding to the fermentation period following nitrogen supplementation of the nitrogen-limited sluggish fermentation was on the focus of our analysis.

## Methods

### Yeast strain and fermentation media

*S. cerevisiae* wine strain PYCC 4072 [26] was maintained at −80°C in YPD medium, containing yeast-peptone-dextrose (20 g L^-1^ glucose, 20 g L^-1^ peptone and and 10 g L^-1^ yeast extract) and glycerol (15% v/v). Prior to use, cells from frozen cultures were transferred to fresh YPD agar plates (20 g L^-1^ agar), and grown for 24 h at 30°C. The previously described chemically defined basal grape juice medium, mimicking a standard natural must with glucose as the sole carbon source [26] was used with minor alterations, specifically the initial available nitrogen concentration present in the three fermentation media and the use of potassium and sodium tartrate instead of potassium tartrate. The medium used contained (per liter): glucose, 200 g; potassium and sodium tartrate, 5 g; L-malic acid, 3 g; citric acid, 0.2 g; K_2_HPO_4_, 1.14 g; MgSO_4_ 7H_2_O, 1.23 g; CaCl_2_ 2H_2_O, 0.44 g; MnCl_2_ 4H_2_O, 198.2 μg; ZnCl_2_, 135.5 μg; FeCl_2_, 32.0 μg; CuCl_2_, 13.6 μg; H_3_BO_3_, 5.7 μg; Co(NO_3_)_2_ 6H_2_O, 29.1 μg; NaMoO_4_ 2H_2_O, 24.2 μg; and KIO_3_, 10.8 μg; vitamins (myo-inositol, 100 mg; pyridoxine HCl, 2 mg; nicotinic acid, 2 mg; calcium pantothenate, 1 mg; thiamine HCl, 0.5 mg; p-amino benzoic acid, 0.2 mg; riboflavin, 0.2 mg; biotin, 0.125 mg; and folic acid, 0.2 mg). The medium pH was adjusted to 3.7 prior to sterile filtration. Nitrogen was supplied as diammonium phosphate (DAP), as the sole nitrogen source, to facilitate the monitoring of nitrogen consumption. For this purpose, a sterile stock concentrated solution of diammonium phosphate, with pH adjusted to 3.7, was added prior to fermentation or during the process following nitrogen source depletion.

### Preparation of the inoculum and fermentation conditions

The preparation of *S. cerevisiae* PYCC 4072 inoculum and the fermentation conditions followed essentially those described previously [26]. The inoculum was prepared by pre-growing the yeast culture overnight in 100-mL shake flasks containing 2/3 of this volume (66 mL) of synthetic grape juice medium with 320 mg L^-1^ of assimilable nitrogen (corresponding to 1.5 g L^-1^ of diammonium phosphate (NH_4_)_2_HPO_4_), at 25°C and 150 rpm. This preculture was used to inoculate the main cultures with an initial concentration of the viable population of 10^6^ colony forming units (CFU) per milliliter. Fermentations were carried out in 500 mL Erlenmeyer flasks (120 rpm) equipped with cotton stoppers, keeping the ratio volume of grape juice medium:volume of the flask equal to 2/3, at 20°C, mimicking vinification conditions. The effect of the initial nitrogen concentration on yeast performance during fermentation was examined using a basal grape juice medium containing an initial assimilable nitrogen concentration of either 90 mg L^-1^ (424 mg L^-1^ of (NH_4_)_2_HPO_4_) for the nitrogen-limited fermentation (LF) or 320 mg L^-1^ (1.5 g L^-1^ of (NH_4_)_2_HPO_4_) for the complete fermentation (CF). After 72 hours of fermentation of the medium with the limited nitrogen concentration (i. e. approximately 2 days after nitrogen depletion) the sluggish fermentation broth was divided into two 250 mL Erlenmeyer flasks (keeping the ratio volume of grape juice medium:volume of the flask equal to 2/3), and assimilable nitrogen (230 mg L^-1^ as 1.1 g L^-1^ of (NH_4_)_2_HPO_4_) was added to one of the fermentation broths (identified as the refed fermentation - RF) while the other (LF) remained as the control.

### Fermentation monitoring

Yeast growth was monitored throughout fermentation by following culture optical density at 600 nm and by the determination of the concentration of viable cells, based on the number of CFU mL^-1^ grown onto YPD solid medium, incubated for 3 days at 30°C, and the progress of alcoholic fermentation by the measurement of glucose, assimilable nitrogen and ethanol concentrations. Culture supernatants were obtained by centrifugation of cell cultures harvested after 0, 6, 24, 48, 72, 80, 96 and 144 h of fermentation. The ammonium concentration was determined as described before [28] and ethanol and glucose concentrations were determined by high-pressure liquid chromatography (HPLC) using an Aminex HPX87H ion exchange chromatography column, eluted at room temperature with 0.005 M H_2_SO_4_ at a flow rate of 0.6 mL min^-1^ for 30 min, using a refractive-index detector. The retention times for glucose and ethanol were 8.3 min and 19.4 min, respectively. Reproducibility and linearity of the method were tested, and concentrations were estimated based on appropriate calibration curves. The results represent the average values of three independent experiments. Error bars depict standard deviation from the mean.

### Glucose transport assays

Initial glucose uptake rates were determined at different fermentation times using radiolabeled D-[U-^14^ C]glucose (PerkinElmer, MA, USA, 300 mCi mmol^-1^, 11.1 GBq mmol^-1^) as described previously [29,30] with minor modifications. Cells were harvested by centrifugation from the fermentation broths, washed twice with 10 mL ice-cold water and resuspended to a density of 10^9^ cells mL^-1^ in TM buffer (0.1 M MES, 41 mM Tris, pH 5.5). Aliquots of 40 μL of this cellular suspension were transferred to 5 ml Röhren tubes and incubated at 30°C for 5 min for temperature equilibration. After this period, 10 μL of radiolabeled D-[U-^14^ C]-glucose was added to each tube by vigorous vortexing. The final concentrations of radiolabeled D-[U-^14^ C]-glucose in the tubes were 1, 2, 5, 10, 20, 50, 100 and 200 mM. These radiolabeled glucose solutions were prepared by dilution in distilled water of a 1 M radiolabeled D-[U-^14^ C]-glucose stock solution. After 5 seconds of cell incubation with the radiolabeled glucose, reaction was stopped by vigorous quenching with 3.5 mL ice-cold demineralized water. Cells were immediately collected by filtration (Whatman GF/C glass microfiber membranes), the filters transferred to scintillation vials containing 7 mL liquid scintillation cocktail Ultima Gold^TM^ MV (Perkin-Elmer) and the radioactivity measured in a Beckman LS 5000TD scintillation counter. Sugar uptake rates were calculated using results obtained in duplicate for each sugar concentration and each transport assay was repeated at least twice. The obtained data were fitted to Eadie-Hofstee plots using graphPad Prism (GraphPad Software Inc., La Jolla, CA) to identify the presence of one or two glucose uptake systems. The estimation of the kinetic parameters *K*_m_ and *V*_max_ was performed using the Michaelis–Menten kinetic model for one or two carriers. An iterative computation procedure described in [31] was used for modeling the two-carrier system using both Michaelis-Menten and Lineweaver-Burk plots.

### Biclustering analysis for regulatory module identification

Results of the transcriptional response of *S. cerevisiae* PYCC 4072 during the three fermentations performed under conditions similar to those examined in the present work [26] were explored using CCC-Biclustering, a state of the art biclustering algorithm specifically developed for time series gene expression data analysis [27]. Biclustering enables the identification of groups of genes with highly coherent expression profiles in a subset of the time points analyzed (local patterns) without forcing all the genes to belong to one group and allowing genes to belong to more than one group. Groups of genes with coherent expression patterns under all the time points analyzed (global patterns) are also discovered, when they exist. Biclustering should thus be preferred to standard clustering techniques, such as those used in [27], in gene expression data analysis. When clustering is used, all genes are forced to belong to one group (cluster), each gene can only belong to one group, and within-group similarities are computed using all the time points analyzed, thus meaning that only global patterns can be identified. This results either in a large number of groups with a short number of genes, which are difficult to analyze and are in most cases uninteresting from a biological point of view, or in a small number of groups with a large number of genes, where some genes behave coherently and the large majority of the remaining genes in the group have uncorrelated expression profiles, as it is the case of most clusters found in [26]. Moreover, clustering algorithms are not able to deal well with missing values, common in expression data, forcing genes with missing values to be deleted or missing value imputation to be performed prior to the clustering analysis, thus augmenting noise. In [26] missing values were filled before clustering.

CCC-Biclustering finds groups of genes with similar expression patterns in contiguous sets of the time points analyzed, thus discovering local expression patterns that would not be uncovered using a global analysis technique, such as clustering. More specifically, and since the algorithm performs an exhaustive search, all maximal CCC-Biclusters (Contiguous Column Coherent Biclusters), maximal sets of genes with maximal expression profiles in consecutive time points, are discovered. In order to avoid the removal of genes with missing expression values at certain time points and therefore guarantee that potentially relevant genes were not excluded *a priori* from the biclustering analysis, CCC-Biclustering was extended to cope with missing values. The biclusters discovered were then sorted according to the statistical significance (*p*-value) of their expression pattern [27]. The *p*-value gives the probability of random occurrence of a bicluster (group of genes sharing the same expression profile) in a gene expression matrix of a given size.

The analysis was focused on the biclusters containing the hexose transporter encoding genes, particularly in the period following ammonium supplementation of the nitrogen-limited fermentation.

## Results

### Glucose uptake kinetics during alcoholic fermentation under different nitrogen availability conditions

**Figure 1:**
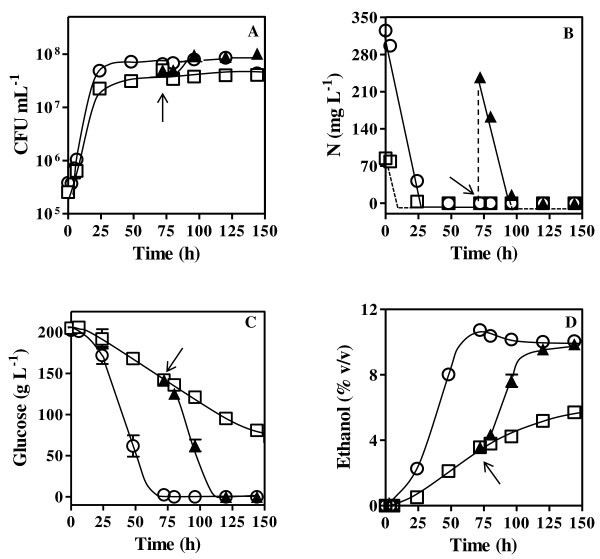
**Growth, nutrient consumption and ethanol accumulation during alcoholic fermentation carried out under different nitrogen availability conditions.** Growth curves and fermentation performance of *S. cerevisiae* PYCC 4072 were followed during 144 h of incubation at 20°C, by determining the concentrations of: (**A**) colony forming units (CFU mL^-1^), (**B**) ammonium, (**C**) glucose and (**D**) ethanol in the fermentation broth. Yeast cells were cultivated in synthetic basal grape juice medium with 200 g L^-1^ glucose and different nitrogen (N) concentrations leading to: i) complete fermentation (CF) [320 mg L^-1^ N] (○); ii) nitrogen-limited fermentation (LF) [90 mg L^-1^ N] (□); iii) nitrogen-limited fermentation LF followed by refed with ammonium after nitrogen exhaustion (RF) [90 + 230 mg L^-1^ N] (▴). The arrow indicates the time of nitrogen supplementation in RF.

The two different initial nitrogen concentrations present in the fermentation media tested in this study led to either a nitrogen-limited sluggish fermentation (LF) (90 mg L^-1^ of assimilable nitrogen) or to a complete fermentation (CF) (320 mg L^-1^ of assimilable nitrogen), this one leading to higher final biomass and ethanol concentrations (Figure1). Even though cell growth stopped in CF medium during the second day of fermentation as the result of nitrogen limitation, all the initial glucose concentration present (200 g L^-1^) was consumed approximately after 3 days of fermentation (Figure1-C). As the result of nitrogen exhaustion during the first hours of the LF fermentation process, the rate of glucose consumption during the rest of the fermentation became much lower when compared with CF. When this sluggish fermentation broth was supplemented with diammonium phosphate (RF), a period of latency, characterized by a non-proliferating yeast viable cell population, was observed during the first hours that followed the supplementation. When yeast growth and fermentation were actively resumed after this period of latency, biomass production and nitrogen and glucose consumption rates reached values similar to those attained in CF (Figure1). Moreover, the ethanol concentration attained during CF and RF was about 10% (v/v) while in LF only 5% (v/v) of ethanol was produced (Figure1-D).

**Figure 2:**
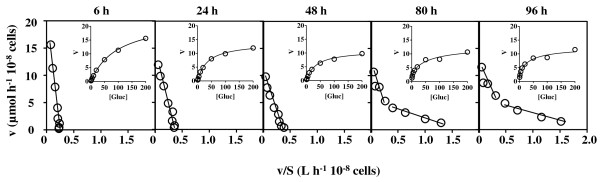
**Eadie-Hofstee and Michaelis-Menten plots of glucose initial uptake rates in CF.***S. cerevisiae* PYCC 4072 cells harvested at the indicated incubation times during the complete fermentation (CF) (Figure1) were used to determine the kinetic parameters *K*_m_ and *V*_max_ depicted in Table1.

**Figure 3:**
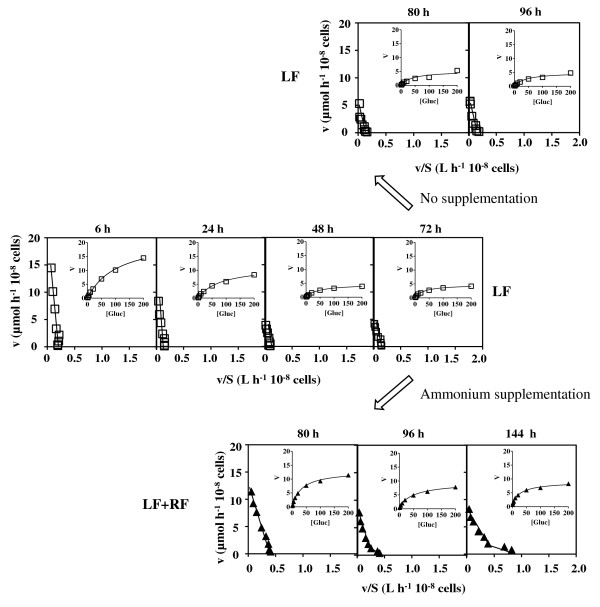
**Eadie-Hofstee and Michaelis-Menten plots of glucose initial uptake rates in LF and RF.***S. cerevisiae* PYCC 4072 cells harvested at the indicated incubation times from the nitrogen-limited fermentation (LF) (Figure1) that was either or not supplemented with diammonium phosphate 72 hours after fermentation start (LF + RF), were used to determine the kinetic parameters *K*_m_ and *V*_max_ shown in Table1.

The values of the kinetic parameters of glucose uptake in yeast cells during the three fermentations under study were determined using radiolabeled D-glucose assays and calculated based on the Eadie-Hofstee and Michaelis-Menten plots (Table1, Figures 2 and 3). Since these glucose uptake kinetic values represent the contribution of one or several co-expressed transporters, with distinct glucose affinities and uptake capacities [17,32], the subsequent analysis of data took into consideration the referred kinetic parameters of the Hxt1-7p transporters when individually expressed [17,18,21,32]. The determination of the kinetic parameters was based on the Michaelis-Menten model that allows, at most, the identification of high- and low-affinity glucose uptake system components. This means that the eventual presence of a moderate-affinity system would not be detected by this analysis. After 6 h of fermentation, when the assimilable nitrogen concentration present in both CF and LF conditions did not limit growth, only slight differences in the kinetic parameters of glucose uptake in cells harvested from both fermentation broths were registered (Table1, Figures 2 and 3). The affinity constant (*K*_m_) values calculated correspond to a low-affinity glucose uptake system such as Hxt1p (*K*_m_ = 100 mM) [17,32]. As the glucose concentration decreases in CF, a gradual decline in the affinity constant is observed. This decline is less marked in LF and, after 24 h of fermentation, an Hxt1p-like affinity constant is still observed. A progressive reduction of the transport capacity (*V*_max_) was registered throughout CF and LF fermentations until the second day of fermentation. Interestingly, the glucose uptake rate, although significantly reduced during sluggish fermentation (LF), was still detectable (Table1, Figure3). Indeed, glucose uptake capacity was not completely abrogated during the extended nitrogen starvation period in LF, remaining approximately 20% of the initial glucose uptake capacity during process monitoring (Table1). After glucose depletion from the CF medium (after 72 h of incubation) it was possible to identify two glucose uptake systems (Table1, Figure2), suggesting the presence of both a low-affinity uptake system (*K*_m_ = 67.5 ± 14.9 mM or *K*_m_ = 45.2 ± 10.2 mM) and a high-affinity uptake system (*K*_m_ = 2.1 ± 0.6 mM or *K*_m_ = 1.9 ± 0.3 mM) after 80 or 96 h of fermentation, respectively. The supplementation of LF with ammonium following nitrogen exhaustion resulted in the stimulation of glucose consumption rates (Figure1-C), consistent with the registered increase of glucose uptake capacity eight hours following ammonium refed. During this period, the affinity for glucose, characteristic of a low-affinity uptake system, maintained essentially the values registered in LF cells before ammonium supplementation. The low-affinity glucose transporter was detected during RF, similarly to CF, but contrarily to CF the presence of the high-affinity glucose uptake system in yeast cells from RF was detected even when glucose-repressible concentrations were still present in the fermenting medium (*K*_m_ = 2.7 ± 0.4 mM, for cells harvested after 96 h of fermentation) (Table1, Figure3). 

**Table 1:** **Kinetic parameters*K***_**m**_**and*V***_**max**_**of glucose uptake in yeast cells harvested during the three fermentation curves examined in this study (Figure**1**) corresponding to different nitrogen supplementations of the fermentation medium: complete fermentation (CF), nitrogen-limited fermentation (LF) and refed fermentation (RF)**

**Incubation Time (h)**	***K***_**m**_**(mM)**			***V***_**max**_**(μmol h**^**-1**^**10**^**-8**^**cells)**		
	**CF**	**LF**	**RF**	**CF**	**LF**	**RF**
**6**	99.2 ± 6.5	108.5 ± 10.4		23.2 ± 0.7	21.9 ± 1.2	
**24**	36.6 ± 3.5	81.7 ± 9.4		13.9 ± 0.4	11.5 ± 0.6	
**48**	34.2 ± 4.4	43.8 ± 5.6		11.0 ± 0.5	4.7 ± 0.2	
**72**	ND	31.5 ± 2.9	AS	ND	4.8 ± 0.1	AS
**80**	67.5 ± 14.9	38.9 ± 6.6	33.2 ± 3.3	9.1 ± 0.8	5.1 ± 0.6	12.9 ± 0.4
	2.1 ± 0.6			3.5 ± 0.1		
**96**	45.2 ± 10.2	39.2 ± 8.6	58.2 ± 9.1	8.6 ± 0.7	4.9 ± 0.8	8.7 ± 0.8
	1.9 ± 0.3		2.7 ± 0.4	3.8 ± 0.2		1.0 ± 0.1
**144**	ND	ND	33.1 ± 3.5	ND	ND	7.3 ± 0.4
			1.3 ± 0.3			1.3 ± 0.2

ND: Not determined.

AS: Ammonium supplementation.

### Biclustering analysis of the transcription profiles of yeast cells following ammonium supplementation of a sluggish fermentation

**Figure 4:**
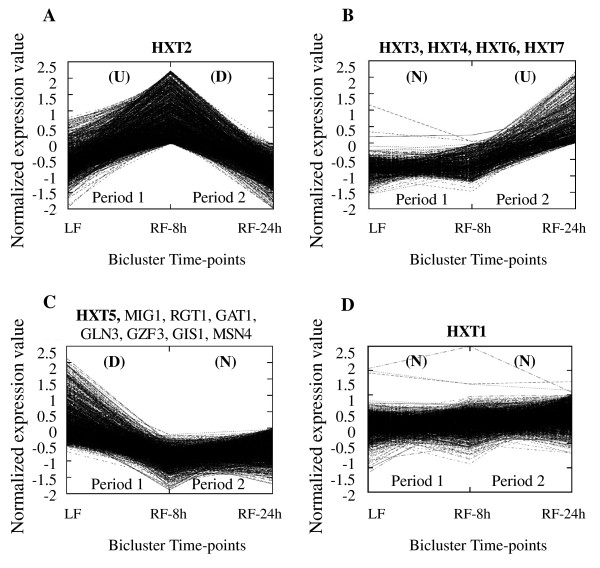
**Patterns of the CCC-Biclusters describing the transcriptional changes occurring in hexose transporter genes (in bold) and selected transcription factors following ammonium supplementation (RF) to the nitrogen-limited fermentation (LF).** Two incubation time periods were compared following 80 h of LF fermentation (a control time point): i) after 8 h of incubation following ammonium supplementation (period 1) and ii) after a period of incubation extending from 8 to 24 h following ammonium supplementation (period 2). The transcriptional alterations during the selected periods are shown under parenthesis as U (Upregulation), D (Downregulation) and N (No changes registered). The full list of genes present in each bicluster is provided in Additional file 1.

In order to get clues into the Hxt transporters that may be responsible for glucose uptake in the recovery of glucose uptake rates following ammonium supplementation of LF, a biclustering analysis was applied to the microarray data from the transcriptional response of *S. cerevisiae* PYCC 4072 cultivated under different nitrogen availability conditions similar to those examined in our study [26]. The biclusters containing the hexose transporter encoding genes *HXT1-7* are shown in Figure4. The full list of the genes belonging to each of the selected biclusters is available in Additional file 1. Results from the biclustering analysis carried out indicate that *HXT2* was the sole glucose transporter encoding gene found to be upregulated during the period of growth latency observed up to 8 h of incubation following ammonium refed (Figure4-A, period 1). Since during this period, glucose concentration was above 100 g L^-1^ the increase of *HXT2* transcription was not expected based on previous reports on the repression of this gene transcription by Mig1p and Rgt1p under glucose repressing concentrations [14]. However, consistent with this observation, the transcription of *MIG1* and *RGT1* genes was found to be downregulated during the same period of time (Figure4-C). Moreover, *HXT2* was also previously found to be induced during the small lag phase characteristic of the start of alcoholic fermentation and associated to growth resumption [33]. The transcription level of the genes coding for Hxt3p, Hxt4p, Hxt6p and Hxt7p remained unchanged during the early response to ammonium supplementation, but exhibited a significant increase during the period of intense glycolytic and fermentative activities following growth resumption in the ammonium supplemented broth (Figure4-B, period 2). This evidence is in agreement with the identification of a glucose uptake kinetics composed of both a low- and a high-affinity glucose uptake system during the same period of fermentation. However, similarly to *HXT2*, the increase of *HXT4HXT6* and *HXT7* transcription levels when glucose-repressible concentrations were present in the external medium was not expected [14,15], suggesting a general derepression of these glucose-repressible HXT genes during this phase. Only *HXT5*, coding for a moderate-affinity glucose transporter, was found to be downregulated following the addition of ammonium to the sluggish fermentation (Figure4-C, period 1). Moreover, an Hxt5p-like kinetics could not be detected during the nitrogen-starved fermentation. Based on the information gathered in the YEASTRACT database dedicated to potential regulatory associations between transcription factors and genes [34], which considers both the documented evidences and the presence of binding sites in the promoter sequences of a particular gene, it was possible to identify transcription factors putatively involved in *HXT5* transcription, as suggested by the transcription profile similar to the one for *HXT5*. These include the nitrogen catabolite repression regulators Gat1p, Gln3p and Gzf3p [35], Gis1p, involved in the upregulation of several genes upon nutrient starvation [36] and Msn4p, involved in activation of stress responsive genes [37]. This association is consistent with the reported activation of *HXT5* transcription during nitrogen starvation [21] and the presence of STRE and HAP elements in *HXT5* promoter regions [38]. The transcription level of the low-affinity glucose transporter encoding gene *HXT1* was not altered in response to the addition of ammonium (Figure4-D) and an Hxt1p-like kinetics for glucose uptake in yeast cells following ammonium supplementation was not either detected.

## Discussion

The kinetics of glucose uptake and the expression and activity of the different hexose uptake systems of *S. cerevisiae* are considered essential to industrial alcoholic fermentation performance. However, the dynamics of glucose uptake kinetics during the different stages of fermentation, depending on glucose and nitrogen availability, is very poorly characterized. During the present study, it was shown that glucose transport capacity is maximal during the early stages of fermentation, when nitrogen concentration did not limit growth both in CF and LF media. Under such conditions, glucose transport is essentially sustained by the low-affinity and high-capacity glucose transporter Hxt1p, as suggested by the high *K*_m_ and *V*_max_ values calculated [17,32]. It is known that in the presence of high glucose concentrations available at the beginning of fermentation (above 100 g L^-1^), *HXT1* transcription is induced by the coordinated activity of the Snf3p/Rgt2p-Rgt1p and HOG pathways [33,39]. As the nitrogen concentrations available became a growth limiting factor and glucose concentrations decreased, the values calculated for the *K*_m_ for glucose decreased to values close to those calculated for the low-affinity glucose transporter Hxt3p when individually expressed (*K*_m_ = 30–60 mM) [17,32]. The absence of an Hxt1p-like uptake kinetics after nitrogen depletion is consistent with the fact that, under nitrogen starvation, the induction of *HXT1* transcription by glucose is repressed and the protein is degraded via the TOR pathway, which controls growth and metabolism in response to nutrient availability [40,41]. After the addition of ammonium to a nitrogen-limited fermentation containing *HXT1*-inducible sugar concentrations, the expected glucose uptake kinetics characteristic of Hxt1 was still not observed, consistent with the lack of variation in *HXT1* transcription level following nitrogen supplementation. The low-affinity glucose transporter Hxt3p was reported to remain functional as long as glucose is available in the fermentation medium [14], but, in this study, we found kinetic evidences that Hxt3p is functional even in the absence of glucose, when ethanol concentrations reached maximum values (10% (v/v)) or during sluggish fermentation. Remarkably, the transcriptional response of cells of the same yeast strain, cultivated under different nitrogen availability conditions similar to those examined in our study, showed a remarkable upregulation of *HXT3* at later fermentation stages presumably associated to the response to high ethanol concentrations [26,42]. Results from our study suggest that Hxt3p expression is not necessarily abrogated by glucose, or by nitrogen limitation, playing a major role throughout both fermentations, independently of the initial conditions tested. Specifically during the period of growth latency that followed ammonium supplementation, the glucose uptake kinetics revealed that the affinity for glucose remained unchanged and characteristic of a low-affinity glucose transporter such as Hxt3p, but that the transport capacity increased, suggesting that new glucose transporters had been synthesized. Although the results from the biclustering analysis indicated an increase in the transcription level of the moderate/high-affinity glucose transporter encoding gene *HXT2* during the lag phase registered after ammonium refed, an affinity constant characteristic of Hxt2p could not be detected during this period. Following this period, the genes *HXT3, HXT4HXT6* and *HXT7* coding for hexose transporters with distinct affinities for glucose were found to be transcriptionally activated, but kinetic evidences were only obtained for the presence of Hxt3p and Hxt6/7p. Although the activity of glucose transporters encoding genes is highly regulated by glucose concentration, the suggested derepression of glucose-repressible genes under the experimental conditions tested in this study can be associated to the decrease in *MIG1* and *RGT1* transcription level registered following ammonium refed. Also, the fact that plasma membrane H^+^-ATPase activity is activated by ethanol to counteract ethanol toxicity [43,44], and that ethanol concentration significantly increase in RF broth after ammonium supplementation, the expression of different glucose uptake systems at this phase is a way to increase glycolytic flux and ATP production to support the requirements of an active plasma membrane H^+^-ATPase. It is known that ethanol leads to the transcriptional activation of *HXT6* and *HXT7*[45,46]. In the analysis of the results of glucose uptake kinetics we should have missed the identification of transporters with intermediate affinities such as those corresponding to the moderate/high-affinity glucose transporters Hxt4p and Hxt2p and of the moderate-affinity glucose transporter Hxt5p. Moreover, further research is needed to establish the presence and activity of Hxt2p and Hxt4p following ammonium supplementation. Nevertheless, the genes *HXT1-HXT7* are, among a family of eighteen hexose permeases encoding genes, those that exhibit the highest transcript levels throughout the three fermentation conditions tested in our study [26].

## Conclusions

There is a generalized idea that the main reason for stuck and sluggish alcoholic fermentations under nitrogen-limited conditions is the inhibition of sugar uptake as the result of the strong reduction of the activity of sugar transporters, triggered by protein synthesis arrest and transporters degradation. Although the glucose uptake capacity of yeast cells does indeed suffer a reduction during nitrogen starvation (about 80% of initial capacity), the glucose uptake was not eliminated and it was presumably maintained essentially by Hxt3p. Results also indicate that after ammonium supplementation, the recovery of the nitrogen-starved sluggish fermentation can be achieved by the increase of glucose uptake capacity, presumably related to the *de novo* synthesis of new transporters. This hypothesis was suggested by the transcription activation of *HXT2* during the period of growth latency following ammonium supplementation and, subsequently, by the co-expression of Hxt3p, Hxt4p, Hxt6p and Hxt7p encoding genes, accompanying active fermentation recovery. Results of the glucose uptake kinetics also suggest the presence of glucose transporters with different glucose affinities and capacities, such as those exhibited by Hxt3p and Hxt6/7p. Results from this work provide new insights into the kinetics of glucose uptake in yeast cells throughout different fermentations, suggesting the combined effect of the presence of different nitrogen and glucose concentrations on the expression of HXT transcripts. For this reason, this study is useful to guide the development of more rational strategies to improve yeast performance when exposed to nutrient fluctuations and/or stressful conditions during alcoholic fermentation.

## Competing interests

The authors declare that they have no competing interests.

## Authors’ contributions

MP performed and monitored alcoholic fermentations, glucose uptake assays and contributed to the analysis of the results and the writing of the manuscript under the scientific supervision of IS-C who conceived and coordinated the study. SCM performed the CCC-biclustering analysis. AM-F participated in the design of fermentation experiments and in discussions. All authors read and approved the final manuscript.

## Supplementary Material

Additional file 1 ** CCC-Biclusters of the transcriptional changes occurring following ammonium supplementation (RF) to the nitrogen-limited fermentation (LF).** Two incubation time periods were compared following 80 h of LF fermentation (a control time point): i) after 8 h of incubation following ammonium supplementation (period 1) and ii) after a period of incubation extending from 8 to 24 h following ammonium supplementation (period 2). The transcriptional alterations during the selected periods are shown under parenthesis as U (Upregulation), D (Downregulation) and N (No changes registered). Click here for file
